# Explicit Expressions for a Mean Nanofibre Diameter Using Input Parameters in the Process of Electrospinning

**DOI:** 10.3390/polym15163371

**Published:** 2023-08-11

**Authors:** Petr Filip

**Affiliations:** Institute of Hydrodynamics, Czech Academy of Sciences, 160 00 Prague, Czech Republic; filip@ih.cas.cz

**Keywords:** electrospinning, nanofibre diameter, explicit relations

## Abstract

The process of electrospinning is subject to a variety of input parameters ranging from the characterization of polymers and solvents, the resulting solutions, the geometrical configuration of the device, including its process parameters, and ending with crucial parameters such as temperature and humidity. It is not possible to expect that functional expressions relating all these parameters can be derived in a common description. Nevertheless, it is possible to fix the majority of these parameters to derive explicit relations for a restricted number of entry parameters such that it contributes to the partial elimination of the classical trial-and-error method saving time and financial costs. However, several contributions providing such results are rather moderate. Special attention is provided to fibre diameter approximation as this parameter strongly influences the application of nanofibrous mats in various instances such as air filtration, tissue engineering, and drug delivery systems. Various difficulties connected with the derivation of these explicit relations are presented and discussed in detail.

## 1. Introduction

**Figure 1:**
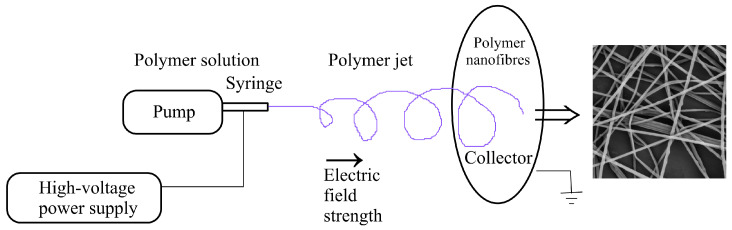
A simplified definition sketch of the process of electrospinning.

Electrospinning represents a very efficient and relatively cheap method of producing polymeric (nano)fibres. Currently, their application, originally in air filtration, covers various branches from water desalination to tissue engineering and drug delivery systems. Figure 1 illustrates an example of electrospinning processes where a motionless device is used, and the spinning jets of polymer solution are evoked by the electric field generated by a high-voltage power supply. The viscoelastic jets emanate from the so-called Taylor cones [1,2] appearing on the surface of the polymer solution due to an applied high voltage and during the passing of the jets to a collector, first straightforward and later in a non-bifurcating spiral path, the used solvent evaporates and hence, the (nano)fibres solidify and deposit on the collector. This relatively simple process is in fact very complex and made complicated due to the possibility of extensive combinations of participating entry parameters [3,4,5,6].

The number of input parameters in the process of electrospinning are relatively high and these parameters can be roughly sorted into five categories:

- Properties of the used polymer(s) (molecular weight, molecular weight distribution, and topology of macromolecules);
- Properties of the used solvent(s) (surface tension, solubility parameters, and relative permittivity);
- Properties of the prepared solutions (concentration, viscosity, viscoelasticity, and specific conductivity);
- Characteristics of the experimental setup (electric field strength, tip-to-collector distance, polarity, geometrical arrangement of the collector, needle diameter, and flow rate);
- Environmental characteristics (temperature and humidity).

Despite the incompleteness of this list, it is apparent that a description of mutual interplay among all these parameters cannot be achieved. On the other hand, there exist n-tuples of parameters mutually interlaced as for instance concentration-viscosity-fibre diameter. The impact (weight) of the individual characteristics is not at the same level as can be documented by a significant dominancy of humidity [7].

It seems that the overall complexity of the process of electrospinning can be analysed by fixing the majority of parameters for a chosen polymeric material and exploring the relationships among those selected. This approach is chosen in practically all contributions and can be further subdivided into two groups: (a) derivation of the general qualitative dependencies (mutual behaviour between the analysed parameters, increasing, decreasing, etc.); (b) and a determination of the explicit relations.

Knowledge of the range within the first group of parameters assists to decide how to better align the individual parameters for achieving the required result, such as for example a mean nanofibre diameter. However, an approximation is based on a trial-and-error approach. The explicit relations ranging to the second group should noticeably eliminate successive approximation and hence, it should result in apparent time saving and a reduction in financial costs.

In the literature, the explicit determination of individual parameters dependent on the selected ones can be classified to approximately three groups:

- Linear and power-law relations;
- Quadratic (polynomial) relations;
- Other approaches.

The functional (empirical or phenomenological) expression relating the individual parameters should comply with the following attributes:

- Relatively simple algebraic form;
- A minimum of adjustable coefficients;
- Mutual unanimous determination of the individual coefficients;
- ’Robustness’ of the coefficients;
- Possible physical interpretation of the coefficients;
- A number of valid figures in the numbers representing the coefficients should comply with experimental errors, very often the indicated number of figures contradicts experimental accuracy.

Generally, a higher number of adjustable coefficients create a higher probability that the coefficients can influence each other. This phenomenon can result in four adverse factors:

- Non-uniqueness of the values of the coefficients;
- Existence of more n-tuples with comparable approximation;
- Improper physical interpretation;
- Addition of more experimental points can significantly change the existing values of the coefficients (decline from robustness).

The item of robustness is closely related with the supposed functional form of the proposed expressions. For instance, a polynomial approximation is closely related with the Weierstrass theorem [8] stating that every continuous function defined on a closed interval [a, b] can be uniformly approximated as closely as desired by a polynomial function, accuracy of an approximation is interlaced with an increasing degree of the polynomial. As the individual powers do not form an orthogonal basis, every change in a polynomial is accompanied by a change in the individual coefficients. The sequence ‘accuracy—degree of a polynomial—a number of coefficients’ generates a compromise concerning the number of adjustable coefficients. There is also a tendency to improve accuracy by including the so-called mixed terms (multiplication of two parameters of the system).

This topic is also closely connected with the notions of approximation and interpolation. While an interpolation represents the strict respecting of the experimental values, i.e., a passage of the model curves through the measured points or only negligible deviation, an approximation is based more on a functional description of real tendencies (monotonicity, increase, decrease, inflection, supremum, and infimum). This approach has two advantages over an interpolation: (1) a more reliable picture relating the parameters, (2) and respecting experimental inaccuracy of the measured characteristics. It is possible to demonstrate with an example of the mean diameter of electrospun nanofibres. Its value for any polymeric material is only approximate with a relatively high standard deviation (appr. ±20%) [7,9]. Hence, it has sense to determine functional courses and not an exact interpolation of the individual inaccurate points.

The aim of this contribution is to summarize present approaches to the explicit modelling of the parameters in the process of electrospinning and to introduce pros and cons of the individual approaches. An emphasis is paid to the determination of the mean nanofibre diameter.

## 2. Modelling and Discussion

A number of the parameters are relatively high [10]. Their importance is subject to the intensity with which their relatively small changes can influence the whole process of electrospinning. The result can range from complete failure of the process of electrospinning (changes in humidity) to modifications of a diameter of electrospun nanofibres or morphological structure. As already introduced above, an explicit description of the parameters can be roughly divided into three categories, which are consecutively discussed below.

### 2.1. Linear and Power-Law Relations

**Figure 2:**
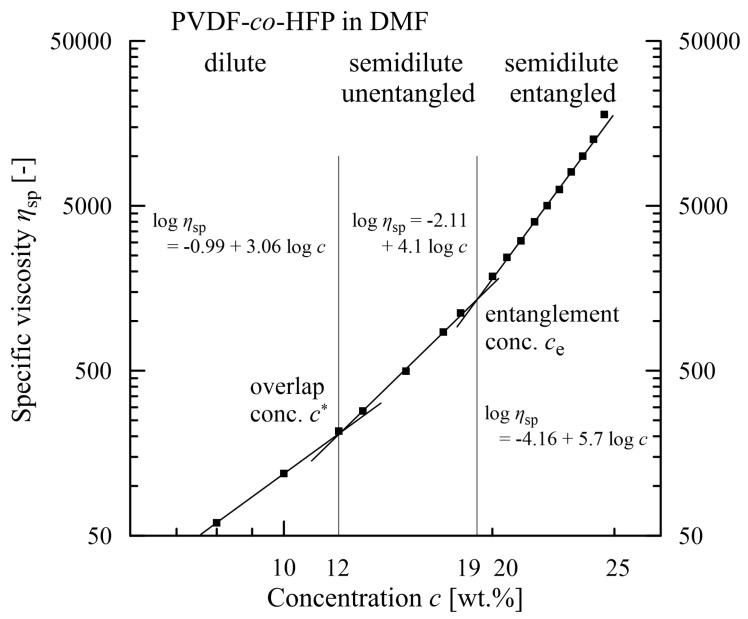
Evaluation of the overlap and entanglement concentrations for poly(vinylidene-*co*-hexafluoropropylene) solved in N,N’-dimethylformamide [17].

Usually, for better clarity, only two parameters are taken into account, much less often three or even more. However, information acquired from only the dependencies between two parameters can be very beneficial, as for instance a dependence of specific viscosity *η*_sp_ (relating viscosity of polymer *η*_0_ and of solution *η*_s_: *η*_sp_ = (*η*_0_ *– η*_s_)/*η*_s_)) on polymer concentration. The slopes of a linear segment approximating the courses of this relationship (log (*η*_sp_) vs. log (concentration)) differ in the regions with different concentrations: dilute, semi-dilute unentangled, semi-dilute entangled, and concentrated [11], see Figure 2. The first point of intersection corresponds with the so-called overlap concentration c* (a reciprocal value of the intrinsic viscosity) characterized by an onset of contacts among polymer coils. The successive point of intersection called the entanglement concentration *c*_e_ corresponds to behaviour exhibiting viscoelastic character. The onset of a bead-free morphology of the nanofibres is approximated from below by an expression k.*c*_e_, where a value of the coefficient k is subject to the choice of the individual polymeric materials. This example documents usefulness even if only two parameters are related by the corresponding explicit relations [12,13,14,15,16,17].

In modelling between two parameters *p*_1_ and *p*_2_, only two functional dependencies are dominantly applied. Either a linear relation (sometimes with an offset term), if the normal coordinates are used, or a power-law relation.

*p*_1_ = a_1_ + a_2_ × *p*_2_, (1)

(2) $$p_{1}=a_{3}+p_{2}^{a_{4}}$$

where a_i_, i = 1,...,4, are the adjustable coefficients.

**Figure 3:**
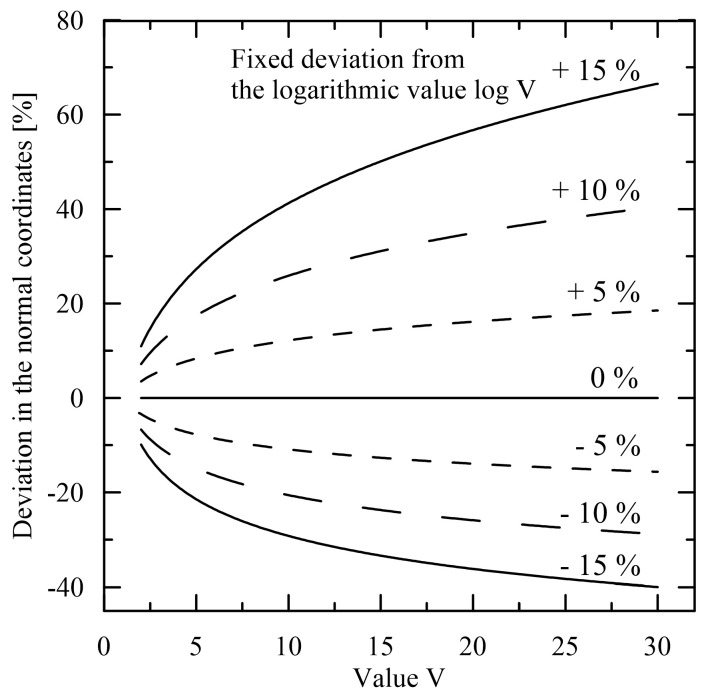
Differences in the deviations expressed in the logarithmic and normal coordinates.

However, the power-law relation (2) is often converted to the log-log coordinates, where a power-law relation is again transformed to the linear relation. Nevertheless, this transformation is rather treacherous as the coefficients optimised in the log-log coordinates have no physical meaning and seemingly good (from the visual viewpoint) approximation in the log-log coordinates can be unacceptable in the normal coordinates. This situation is depicted in Figure 3, where for instance the declared deviation up to 10% in the log-log coordinates (the same is valid for the semi-log coordinates) corresponds to much higher deviations in the normal coordinates. For the value *V* = 30, the deviations attain −29% (≅21.35) and +41% (≅42.15), which corresponds to ±10% for log *V* (≅1.477). The dispersion of the limiting values seems to be rather unattractive from the viewpoint of successful modelling. This implies that only relatively very precise approximation in the log-log or semi-log coordinates can be taken into account.

The diameter of the resulting nanofibres is usually the crucial parameter in potential industrial applications. Hence, strong attention has been paid to the relations of a mean diameter to other parameters. It is necessary to mention that a mean value is very often accompanied with relatively high standard deviation. The variety of obtained diameters for selected materials including their dispersion is summarised in [18].

In [19], the effects of 13 material and operating parameters on electrospun fibre diameters are consecutively modelled by linear (dominantly) and power-law relations. Not in all diameter vs. one parameter relations such modelling seems to be optimal and a more complex description of functional dependencies is inevitable.

**Figure 4:**
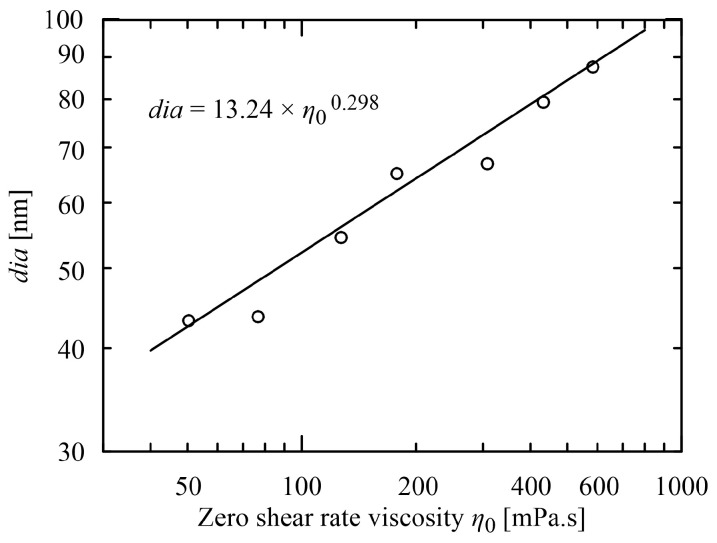
Power-law dependence of nanofibre diameter *dia* on zero shear rate viscosity *η*_0_, experimental data and relation taken from [16].

**Figure 5:**
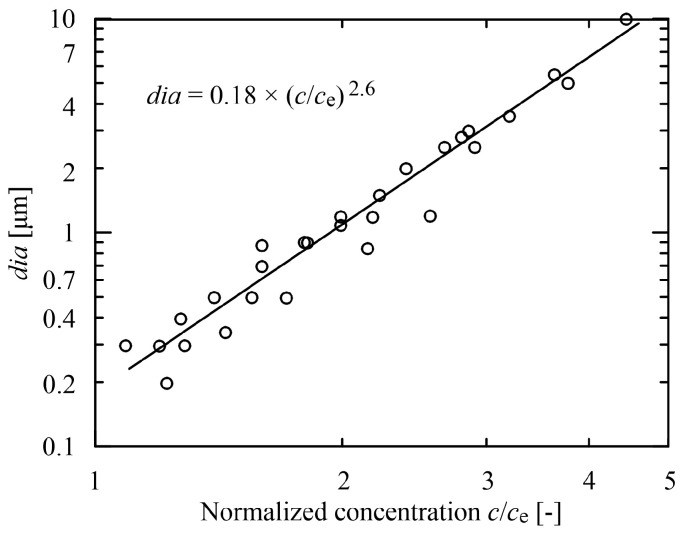
Power-law dependence of nanofibre diameter *dia* on the concentration *c* normalized by entanglement concentration *c*_e_, experimental data and relation taken from [12].

Applying the relations (1,2), the adjustable coefficients strongly depend on the used polymeric material. The explicit relations [12,13,14,15,16,20,21]

(3) $$dia=a_{1}\;\eta_{0}^{a_{2}}$$

between the mean diameter *dia* and the zero shear rate viscosity *η*_0_ were proposed, see Figure 4, and analogously the power-law relation between the mean diameter and the concentration *c* normalized by entanglement concentration *c*_e_ [12,16,22] (see Figure 5).

(4) dia=a1(cce)a2

Based on these two relations it is possible to expect the same relation [22,23,24] between the mean diameter and the dimensionless Berry number *Be* = [*η*].*c*

(5) $$dia=a_{1\;}([\eta ].c{)}^{a_{2}}$$

where [*η*] is the intrinsic viscosity

(6) $$\left[\eta \right]=\underset{c\to 0}{\lim}\frac{\eta_{sp}}{c}$$

The power-law dependence between the mean diameter and the molecular weight *M*_w_ was found in [25]

(7) $$dia=a_{1}(M_{w}{)}^{a_{2}}$$

An influence of the jet diameter *dia*_j_ (through its passage to a collector) on the diameter *dia* of the produced nanofibres was studied in [26,27,28,29,30,31], the power relation between *dia* and *dia*_j_ was proposed in [15]

(8) $$dia=a_{1\;}({dia}_{j}{)}^{a_{2}}$$

Algebraically more complicated relation was proposed [32] for a relation between the mean diameter and simultaneously flow rate *Q* and applied voltage *V*

*dia* = a_1_ + a_2_ × *Q* + a_3_ × *V* + a_4_ × *Q* × *V*. (9)

If either flow rate or voltage is fixed, then *dia* is modelled by the other parameter in a linear way shifted by the offset coefficient.

### 2.2. Quadratic (Polynomial) Relations

Recently, a series of papers have used a quadratic polynomial expression for the evaluation of the mean diameter in the form

(10) $$dia=a_{0}+\sum_{i=1}^{n}a_{i}p_{i}+\sum_{i=1}^{n-1}\sum_{j=i+1}^{n}a_{ij}p_{i}p_{j}+\sum_{i=1}^{n}a_{ii}p_{i}^{2}$$

where *p_i_* represents the *i*-th parameter and n is their total number taken into consideration, a_0_ is the offset coefficient, a_i_ is the linear coefficient, a*_ij_* and a*_ii_* are the mixed and quadratic coefficients, respectively.

If a number of parameters attain 2, 3, or 4, then a number of coefficients correspond to 6, 10 or 15, respectively. This should correspond to a number of experimental points, where their number should be a multiple of a number of considered parameters. In fact, using the relation (10), it is supposed that if the remaining (n − 1) parameters are fixed, the mean diameter can be approximated by a quadratic function of each parameter *p*_i_

(11) $$dia=a_{0}+a_{i}p_{i}+a_{ii}p_{i}^{2}$$

with 3 coefficients a_o_, a*_i_*, and a*_ii_*. There is a question whether, under presence of so many coefficients, each mixed coefficient a*_ij_* is based on the physical grounds or only serves for better approximation of the mean diameter as the parameters *p*_i_ and *p*_j_ can be mutually entirely independent.

Using rel. (10) for n = 2, Mirtic et al. [33] expressed the mean diameter through conductivity and viscosity, and through storage and loss moduli. For n = 3, specifically applied voltage, tip-to-collector distance and concentration, the corresponding expressions were presented in [34,35,36]. Dependence of the mean diameter on 3 parameters (respecting an addition of PEO for improving electrospinning process) is provided in Mirtic et al. [33]. Broumand et al. [37] (n = 2) and Sarlak et al. [38] (n = 6) significantly used the Response Surface Methodology [39,40] in processing the coefficients in rel. (10), i.e., their reduction to a minimum.

### 2.3. Other Approaches

**Figure 6:**
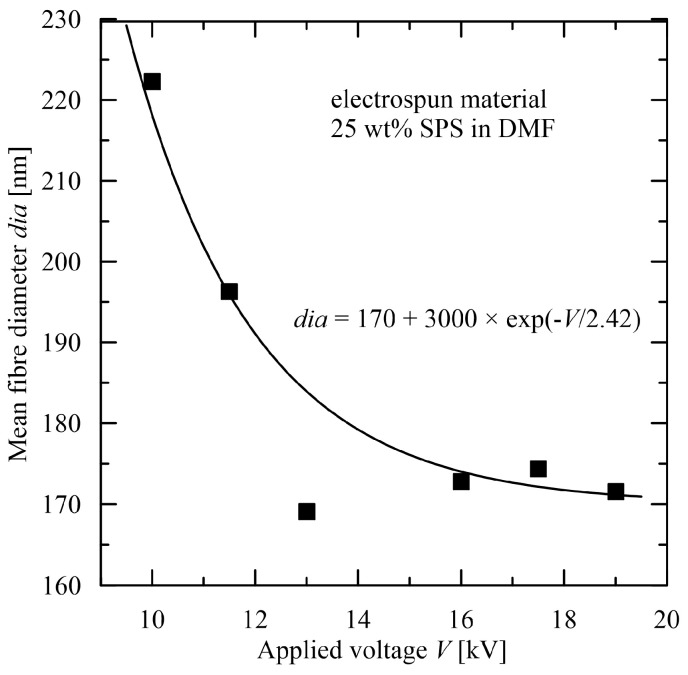
Dependence of mean fibre diameter on applied voltage, experimental data taken from [42] (electrospun material: 25 wt% highly sulfonated polystyrene in N,N-dimethylformamide).

A course of the mean diameter in dependence on various parameters cannot be covered for all materials by a parabolic profile (by a quadratic expression) or its linear simplification. For instance, an application of increasing voltage first results in a decrease in the mean diameter, but is followed by insignificant changes with subsequent increase in voltage [41,42]. Such course is no longer describable with a quadratic term and the following expression is proposed (see Figure 6)

*dia* = a_0_ + a_1_ × exp (−V/a_2_). (12)

**Figure 7:**
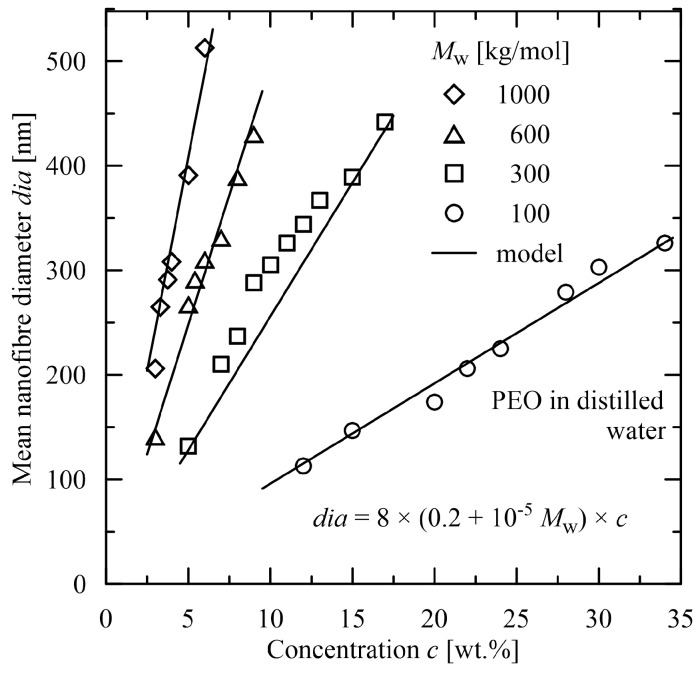
Evaluation of the mean nanofibre diameter in dependence on molecular weight and concentration [43], electrospun material: poly(ethylene oxide) solved in distilled water.

**Figure 8:**
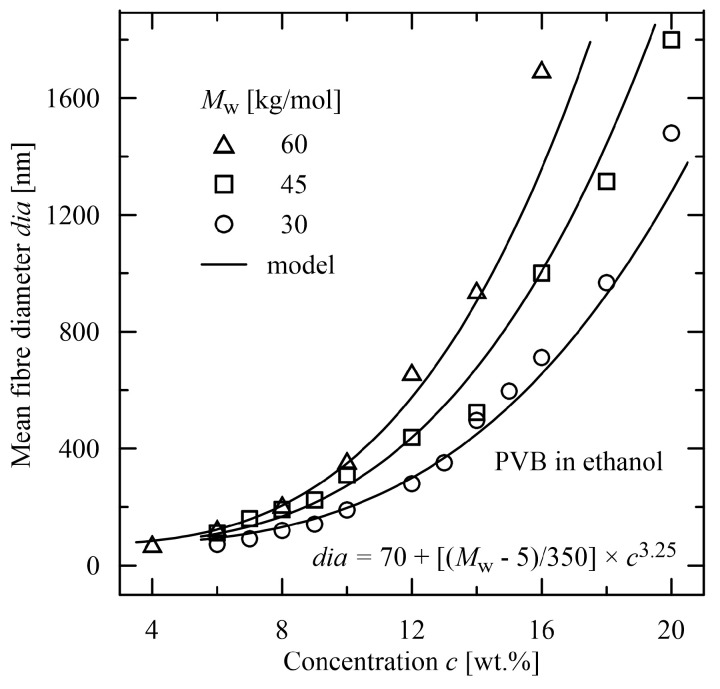
Evaluation of the mean diameter in dependence on molecular weight and concentration [44], electrospun material: poly(vinyl butyral) solved in ethanol.

The advantages of such functions are in their simplicity and, through their Taylor expansions, in a cumulation of polynomial terms. An evident advantage over polynomial expressions is in flexibility with no increase in the number of coefficients. Convenient expressions for a determination of the mean diameter are composed of multiplicative terms, each of them dependent only on one parameter, see Figure 7 and Figure 8. The multiplicative terms in contrast to the additive ones provide a better insight how to adapt the parameters (as molecular weight and concentration in Figure 7 and Figure 8) to obtain the required value of the mean diameter. Such approach covers the whole diagram continuously and interlacing of the individual parameters is evident.

Usefulness of multiplicative terms was also presented in [45], where volume charge density was expressed through a product of powers of parameters (voltage, flowrate, concentration, molecular weight, etc.).

**Figure 9:**
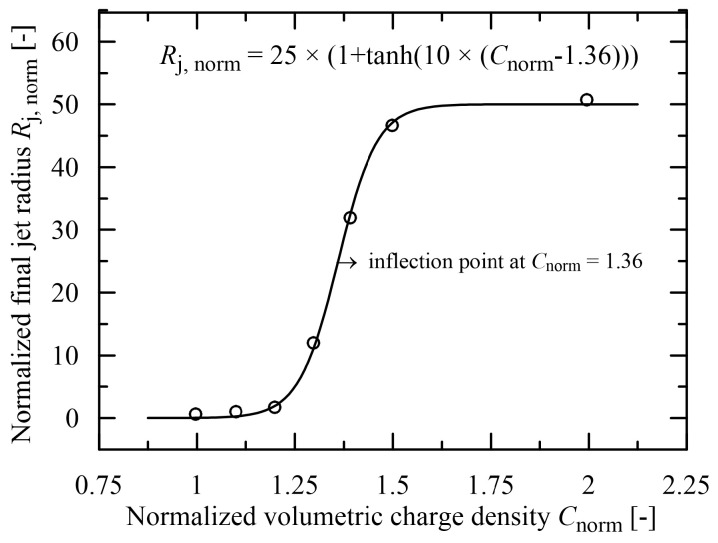
Evaluation of the normalized final jet radius in dependence on normalized volumetric charge density, the experimental data taken from [19].

The achievement of a much higher accuracy has not been connected with the apparent increase in adjustable parameters, as illustrated in Figure 9. A physically more acceptable approximation is achieved with 3 coefficients, only one higher compared with the linear course.

**Figure 10:**
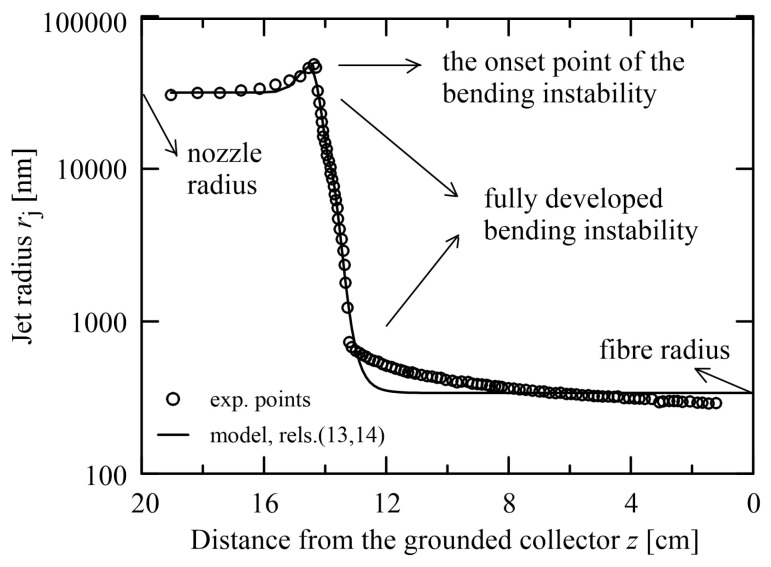
Evaluation of non-monotonous dependence of the jet radius in dependence on the distance from the collector (*z* = 0 corresponds to a collector position), the experimental data taken from [19].

More problems are encountered when a course of the experimental data exhibit non-monotonous behaviour. In such cases, the traditional explicit models and approaches fail since the classical models usually consider monotonous courses. This situation appears in evaluating the jet radius of the viscoelastic jet *r*_j_ between a source and a collector regarding the distance from the collector *z* as presented in [19], see Figure 10. The adequate model curve describes not only the course itself but also provides the onset point of the bending instability. However, in this case the model [46] contains 6 parameters due to non-monotonicity

(13) $$r_{j}=\frac{r_{nozzle\;}e^{-f}+r_{fibre\;}e^{f}}{b+e^{-f}+e^{f}}$$

where

(14) $$f\equiv f\left(z;c,p,q\right)=sign\left(log{\left(cz\right)}^{p}\right).{\left|log{\left(cz\right)}^{p}\right|}^{q}$$

The parameters *r*_nozzle_ (log(*r*_nozzle_) = 4.5) and *r*_fibre_ (log(*r*_fibre_) = 2.53) have a clear physical meaning as depicted in Figure 10, the parameters p (=22), q (=1.4) determine a steep slope and its curving, respectively, the parameter b (=−0.33) determines a measure of non-monotonicity, and the parameter c (=0.162) shifts the curve along the abscissa.

The final fibre diameter terminating the successive diameters of the whipping jet as derived in [28]

(15) $$dia=2\;\times {\left(\gamma \bar{\varepsilon }\;\frac{Q^{2}}{I^{2}}\;\frac{2}{\pi \;\left(2\;ln\chi -3\right)}\right)}^{1/3}$$

is controlled by the flow rate *Q*, electric current *I*, and the surface tension of the fluid *γ*. The symbol $\bar{\varepsilon }$ represents the dielectric constant, χ is the dimensionless wavelength of the instability responsible for the normal displacements. Another formula expressing a dependence of diameter on surface tension is presented in [47].

Modelling of the electrospinning process and a proposal of explicit expressions relating the parameters is more complicated for the polymer solutions, where either solvent or polymeric material are composed of two components. This makes the expressions more complicated. Such situations were modelled in [45] by adding a power term characterizing ethanol concentration (solvent: water + ethanol). A ratio between polymer components (cellulose/poly(ethylene oxide)) [37] was projected into an evaluation of the mean diameter through linear and quadratic terms. A mean diameter generated by electrospinning of a combination of two solvents (N,N’-dimethylformamide (DMF) and acetone) used for a solution of the *co*-polymer poly(vinylidene-*co*-hexafluoropropylene) (PVDF-*co*-HFP) was evaluated by the explicit expression [48]

**Figure 11:**
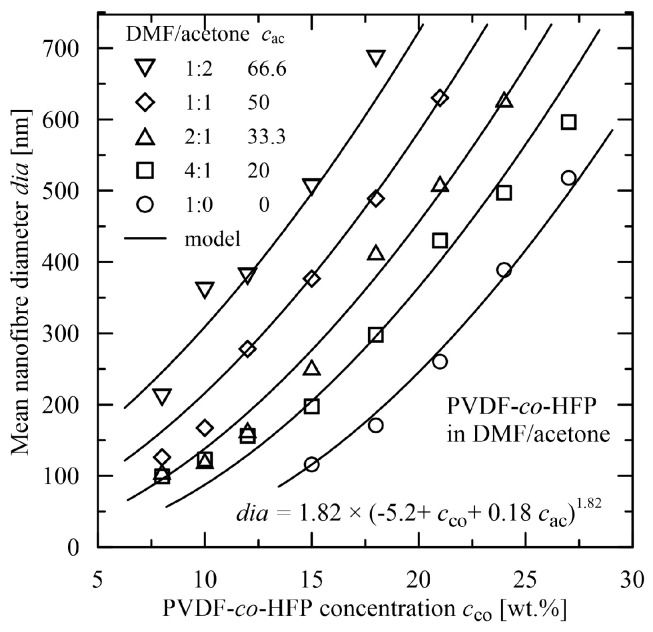
Evaluation of the mean diameter in dependence on *co*-polymer concentration *c*_co_ and a concentration of acetone *c*_ac_ in the solvent (DMF/acetone) [48], electrospun material: PVDF-*co*-HFP solved in DMF/acetone.

*dia* = 1.82 × (−5.2 + *c*_co_ + 0.18 *c*_ac_)^1.82^, (16)

where *c*_co_ is the concentration of the *co*-polymer and *c*_ac_ is the concentration of acetone in the solvent with DMF, see Figure 11.

Recently, an artificial neural network model [49] was developed for the prediction of the mean diameter depending on voltage, flow rate, tip-to-collector distance, and collector rotating speed (the last parameter with negligible impact). The obtained results (a part of the data set used as the tested set) exhibit very good approximation, the open question is with the determination of the functional behaviour of the individual variables.

Based on the above procedures, it is necessary to distinguish between two approaches:

- The aim of the first approach is to evaluate the mean nanofibre diameter in dependence on selected parameters for a specific case. It means to assign a value of the mean diameter to the n-tuple of the chosen coefficients.
- The other approach based on more complicated functional behaviour can be used for altering the diameter. It is possible to determine the n-tuples of coefficients resulting in the same diameter and to choose an optimal n-tuple based on the initial criteria. This approach should work with sufficiently broad ranges of the individual parameters.

## 3. Conclusions

In industrial practice a proper choice of the electrospun nanofibre diameter is usually the significant key factor generating the applicability of final products. This is documented for instance in the following reviews published recently and covering such distant branches as:

- Controlling pollutant emissions before their release into the environment, where membrane diameter governs filtration process [50,51];
- Numerous tissue engineering applications, where the size of nanofibre diameter strongly influences alignment morphology [52];
- Drug delivery systems [53];
- Hydrophobic membranes for oil-water separation [54];
- Tailoring nanofibre diameter for tissue engineered blood vessel scaffold [55];
- Fibrous shape-memory polymer scaffolds, where performance distinctly improves with a reduction in the single fibre diameter [56].

The classical trial-and-error procedure is time consuming and inefficient from the point of view of financial cost. A derivation of explicit functional relations providing the way to influence the diameter through the setting of the individual parameters represents an effective means in handling the whole electrospinning process. To this aim, the application of more complicated algebraic functions replacing the classical linear and quadratic relations seems to be inevitable. On the other hand, a number of adjustable coefficients should be kept at a minimum. As the process of electrospinning qualitatively and quantitatively differs with the materials used, the proposed explicit relations will vary from material to material. However, it is possible to expect that there exist classes of materials describable functionally by the same functional terms with alteration of the adjustable coefficients.

## Data Availability

All experimental data were used from the preceding (cited) literature.
